# Hyaluronic Acid/Ellagic Acid as Materials for Potential Medical Application

**DOI:** 10.3390/ijms25115891

**Published:** 2024-05-28

**Authors:** Beata Kaczmarek-Szczepańska, Konrad Kleszczyński, Lidia Zasada, Dorota Chmielniak, Mara Barbara Hollerung, Katarzyna Dembińska, Krystyna Pałubicka, Kerstin Steinbrink, Maria Swiontek Brzezinska, Sylwia Grabska-Zielińska

**Affiliations:** 1 Department of Biomaterials and Cosmetics Chemistry, Faculty of Chemistry, Nicolaus Copernicus University in Torun, Gagarin 7, 87-100 Torun, Poland; 503555@doktorant.umk.pl (L.Z.); 317323@stud.umk.pl (D.C.); 2 Department of Dermatology, University of Münster, Von-Esmarch-Str. 58, 48149 Münster, Germany; konrad.kleszczynski@ukmuenster.de (K.K.); mholleru@uni-muenster.de (M.B.H.); kerstin.steinbrink@ukmuenster.de (K.S.); 3 Department of Environmental Microbiology and Biotechnology, Faculty of Biological and Veterinary Sciences, Nicolaus Copernicus University in Torun, Lwowska 1, 87-100 Torun, Poland; kdembinska@doktorant.umk.pl (K.D.); swiontek@umk.pl (M.S.B.); 4 Department of Conservation and Restoration of Paper and Leather, Nicolaus Copernicus University in Torun, Sienkiewicza 30/32, 87-100 Torun, Poland; krystynap@doktorant.umk.pl; 5 Faculty of Chemical Technology and Engineering, Bydgoszcz University of Science and Technology, Seminaryjna 3, 85-326 Bydgoszcz, Poland; sylwia.grabska-zielinska@pbs.edu.pl

**Keywords:** hyaluronic acid, ellagic acid, thin films, wound dressing, cutaneous cells

## Abstract

The aim of this work was to develop and characterize a thin films composed of hyaluronic acid/ellagic acid for potential medical application. Its principal novelty, distinct from the prior literature in terms of hyaluronic acid films supplemented with phenolic acids, resides in the predominant incorporation of ellagic acid—a distinguished compound—as the primary constituent of the films. Herein, ellagic acid was dissolved in two different solvents, i.e., acetic acid (AcOH) or sodium hydroxide (NaOH), and the surface properties of the resultant films were assessed using atomic force microscopy and contact angle measurements. Additionally, various physicochemical parameters were evaluated including moisture content, antioxidant activity, and release of ellagic acid in phosphate buffered saline. Furthermore, the evaluation of films’ biocompatibility was conducted using human epidermal keratinocytes, dermal fibroblasts, and human amelanotic melanoma cells (A375 and G361), and the antimicrobial activity was elucidated accordingly against *Staphylococcus aureus* ATCC 6538 and *Pseudomonas aeruginosa* ATCC 15442. Our results showed that the films exhibited prominent antibacterial properties particularly against *Staphylococcus aureus*, with the 80HA/20EA/AcOH film indicating the strong biocidal activity against this strain leading to a significant reduction in viable cells. Comparatively, the 50HA/50EA/AcOH film also displayed biocidal activity against *Staphylococcus aureus*. This experimental approach could be a promising technique for future applications in regenerative dermatology or novel strategies in terms of bioengineering.

## 1. Introduction

For numerous years, the exploration of natural polymers in biomedical contexts has been propelled by their remarkable biocompatibility and biodegradability. Natural polymers or biopolymers are macromolecular compounds synthetized by living organisms or obtained from living organisms [1]. Structurally, they are a sequence of repeating monomers of monosaccharides, amino acids, nucleotides, or esters held by covalent bonds to form polysaccharides, peptides, polyphenols, or polyesters, etc. [2]. The inherent multifunctionality behavior and tunability of natural polymers facilitate their application particularly for various wounds.

To date, hyaluronic acid (HA) is one of the most well-known and commonly used biopolymer in wound dressing applications. It is a high-molecular-weight compound made up of repeating units of *D*-glucuronic acid and *N*-acetyl-*D*-glucosamine linked by alternating β-1,4 and β-1,3 glycosidic linkages [2]. HA plays a crucial role in wound healing by keratinocyte migration, remodeling of the extracellular matrix (ECM), and stimulating fibroblast proliferation. Both hyaluronic acid and its derivatives have undergone extensive testing, individually and in combination with various other compounds, including other biopolymers, phenolic acids, plant extracts, or inorganic additives, for potential medical applications notably in promoting wound healing [3,4,5,6,7]. Phenolic acids are organic compounds, which contain a phenolic ring with hydroxyl and carboxyl groups. They exhibit antioxidant activity and other health benefits, such as the prevention of neoplastic, metabolic, and cardiovascular diseases, as well as osteoporosis, various forms of cancer, and diabetes mellitus [8,9].

Gallic acid (GA), ferulic acid (FA), caffeic acid (CA), or dihydrocaffeic acid (DHCA) have been used as modifiers to hyaluronic acid materials. These phenolic acids exhibit potent antioxidant activity, and materials with their addition are characterized using various analytical techniques to assess their potential for applications in cosmetics, nutraceutical, tissue engineering, and pharmaceutical industries [10,11,12,13].

Ellagic acid (4,4′,5,5′,6,6′-hexahydroxydiphenic acid 2,6,2′,6′-dilactone, EA) is a polyphenol renowned for its anticancer and excellent antioxidant properties, as well as antibacterial activity and UV barrier properties. It is derivative of gallic acid metabolism and is naturally present in fruits, e.g., pomegranates, strawberries, blackberries, raspberries, blueberries, nuts, and green tea [14]. It is effectively used as a modifier in films based on chitosan, apple starch, or sodium alginate targeting food packaging applications [15,16,17], as well as a dietary supplement in a powder, liquid forms, or capsules [17].

From a medical perspective, ellagic acid is known to be combined with chitosan, chitosan/zein, zein, collagen/chitosan, or poly(ethylene glycol) [18,19,20,21,22,23]. Chitosan/ellagic acid films were fabricated using ellagic acid in concentrations of 0, 0.05, 0.1, 0.5, and 1% (*w*/*v*). The earlier results revealed that with the increase in the concentration of the EA, greater surface roughness and hydrophilicity were observed. Additionally, the composite films exhibited increasing amide and ester groups and diffraction peaks of the crystallized ellagic acid. The films were prepared as a potential new clinical approach to cancer treatment [19]. Advanced biological research showed that chitosan/ellagic acid materials could inhibit cancer cell growth in an ellagic acid concentration-dependent manner by inducing apoptosis of cancer cells, as well as suppressing angiogenesis [18]. With regard to zein/ellagic acid-based materials, they were prepared as EA-loaded zein nanoparticles to therapeutic drug delivery systems. Based on the FTIR, NMR, fluorescence spectroscopy, and antioxidant activity measurements carried out by 2,2-diphenyl-1-picrylhydrazyl (DPPH) and 2,2-azinobis-(3-ethylbenzothiazoline-6-sulfonate) (ABTS) assays or antimicrobial activity determination, it can be concluded that the nanoencapsulation of EA boosted its antioxidant and antibacterial activities. This combination of zein and ellagic acid offers a potential strategy against free radicals, as well as providing preventive and treatment option for infections related to Gram-positive and Gram-negative bacteria [20]. In turn, materials based on the chitosan/zein mixture, loaded with ellagic acid, were prepared and characterized as a wound bandaging platform preventing cutaneous-mucous infections, enhancing skin regeneration. The influences of mixture compositions on film properties have been observed where zein presented a significant effect on the release of ellagic acid [21]. Apart from that, collagen/chitosan scaffolds enriched with ellagic acid were developed to manage chronic wounds by reducing generation of reactive oxygen species (ROS). The obtained scaffolds were biocompatible and possessed antioxidant activity. For instance, they supported the recovery of the monkey kidney fibroblast-like COS-7 cell line from the oxidative stress enhanced by UV irradiation, or they exhibited sustained release of ellagic acid in PBS solution [22]. In addition, ellagic acid-loaded poly(ethylene glycol) nanogels were characterized by their capability of scavenging radicals and have been biocompatible with human cervical cancer HeLa cells [23].

Routinely, phenolic acids are used in polymer-based film modification to improve their mechanical properties, as well as surface change. Materials based on polymer, modified with phenolic acids, were prudently characterized by good adhesion and high antioxidant activity [8,24]. Additionally, as we considered above, phenolic acids were successfully implied as additives to biopolymer-based mixtures or pure biopolymers acting as cross-linking agents.

Based on the literature, we established a hypothesis that the hyaluronic acid/ellagic acid film may offer better biocompatibility and a reduced risk of adverse reactions compared with some synthetic substitutes. Derived from natural sources, it is less likely to cause allergic reactions or tissue rejection. The aim of this research was to generate and characterize hyaluronic acid/ellagic acid-based thin films in various HA/EA compositions, 80/20, 50/50, and 20/80, targeting their potential medical applications since the use of ellagic acid as a one of the main films’ components, not a cross-linking agent, seems to be a novel approach in the field of regenerative dermatology with a high applicable impact. Additionally, two different solvents, i.e., AcOH and NaOH, were used to dissolve ellagic acid, and the surface properties of the resultant films were evaluated by atomic force microscopy, contact angle measurements, and antioxidant activity. Finally, ellagic acid release, cytocompatibility, and antimicrobial activity were examined accordingly to present the physicochemical properties of the subjected materials for their potential applications as wound dressings.

## 2. Results

### 2.1. Atomic Force Microscopy (AFM)

**Figure 1:**
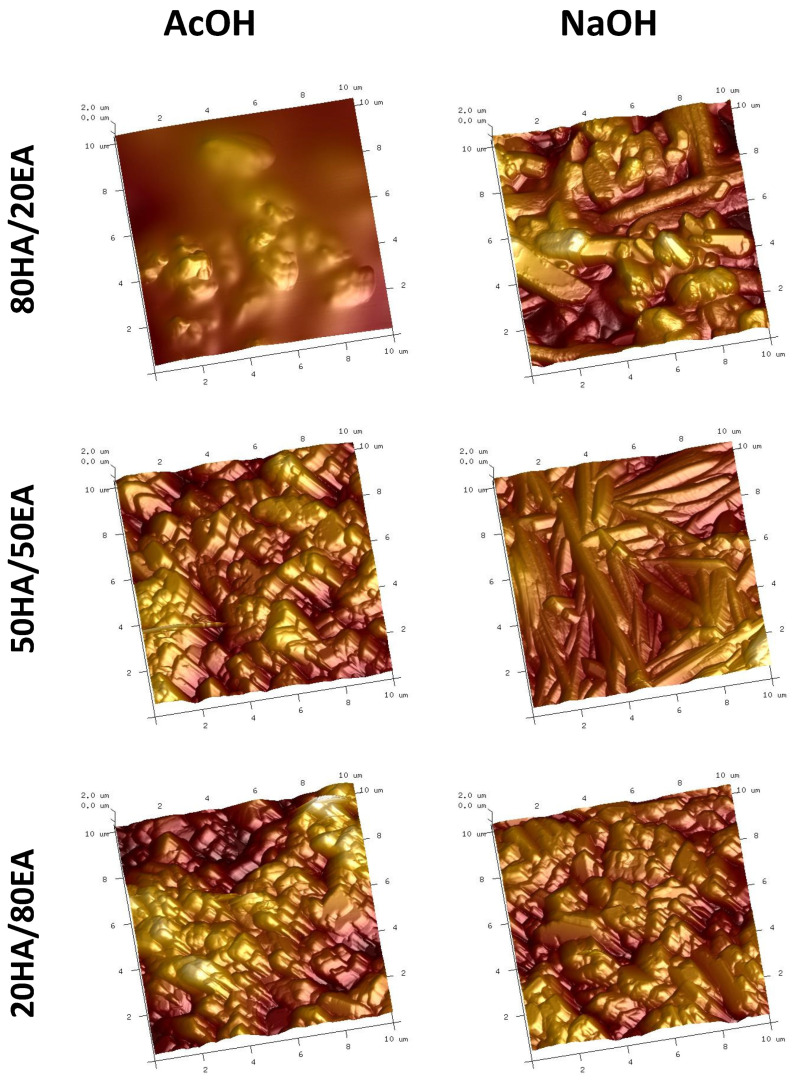
Assessment of AFM images with regard to the various compositions of hyaluronic acid (HA)/ellagic acid (EA) dissolved in either AcOH or NaOH.

The atomic force microscopy (AFM) imaging of the HA/EA films was carried out, and the topography is presented accordingly in Figure 1, while the roughness of the surface was calculated and enclosed in Table 1. Namely, the assessed materials based on hyaluronic acid and ellagic acid dissolved in various solvents were characterized by different roughness parameters where the most significant differences could be observed for 20HA/80EA materials. The films were identified by the highest values of the arithmetic mean roughness (Ra) and the root mean square roughness (Rq) for mixtures where ellagic acid was dissolved in NaOH, even though the images may suggest no significant differences, because visually, the surface morphology looks quite similar. Furthermore, materials composed of 50HA/50EA did not demonstrate statistically significant differences in surface roughness between subjected solvents. Although no significant differences were noticed in the roughness parameters, the surface morphology observed in the images differed visually. Regarding the composition of 80HA/20EA films, those where ellagic acid was dissolved in acetic acid were indicated by higher values of Ra and Rq.

### 2.2. Surface Free Energy

The contact angle measured using two different liquids—glycerine and diiodomethane—surface free energy, and its polar and dispersive components are presented accordingly (Table 2). As can be seen, no possibility to perform measurements was observed for films composed of hyaluronic acid and ellagic acid (dissolved in acetic acid). The drop of glycerine or diiodomethane spread out upon the deposition on the surface. The changes in wettability of the films based on hyaluronic acid with ellagic acid (dissolved in sodium hydroxide) could be observed in comparison with the control sample composed of hyaluronic acid. Films composed of 80HA/20EA/NaOH mixture were characterized by the highest value of the surface free energy dispersive component. No significant difference in the glycerine contact angle between 80HA/20EA/NaOH and 100HA films was observed. Simultaneously, the contact angle for diiodomethane, the surface free energy, and its polar component values were lower than those parameters for hyaluronic acid films. It suggests that 80HA/20EA/NaOH films had lower hydrophilicity than 100HA ones. Furthermore, our results revealed that films composed of 50HA/50EA/NaOH and 20HA/80EA/NaOH were more hydrophilic compared with 100HA, and it could be explained by the fact that their polar components of surface free energy had higher values. The results indicating the most hydrophilic characteristics of the film surface were observed for 50HA/50EA/NaOH materials.

### 2.3. Moisture Content and Antioxidant Activity

The results of the moisture content (MC) study (Table 3) indicated that in subjected materials based on HA/EA/NaOH, the moisture content was distinctly lower when comparing them with the materials containing only HA/EA. This indicated that NaOH should be undoubtedly excluded. Namely, the 80HA/20EA/NaOH film had the highest content of moisture (MC = 12.50 ± 0.93%) in comparison with other HA/EA films. Furthermore, as the content of ellagic acid in the film composition increased, the moisture content decreased. Next, radical scavenging activity (RSA) relevant to the antioxidant activity of the film was also evaluated (Table 3). The DPPH assay presented that all films containing HA/EA, regardless of the used solvent, demonstrated positive radical scavenging activity, unlike control 100HA films. In addition to this, no significant differences in RSA were observed depending on the film composition and the solvent used for ellagic acid dissolution.

### 2.4. Phenolic Acid Release

**Figure 2:**
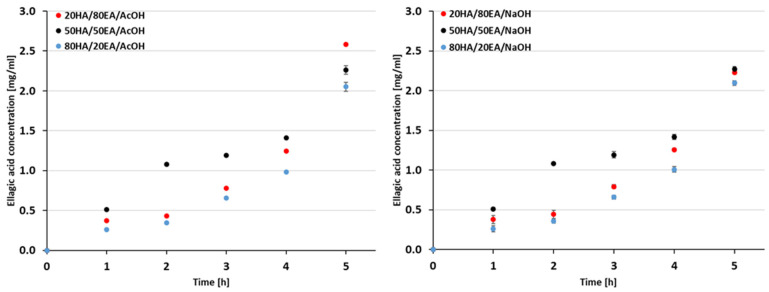
The ellagic acid release from HA/EA films immersed in PBS for 1, 2, 3, 4, and 5 h.

The evaluation of ellagic acid release was carried out for all subjected films and is presented accordingly (Figure 2). We observed a consistent release of ellagic acid from HA/EA films regardless of the used solvent. Specifically, the released concentration remained relatively constant from the first to the fourth hour of incubation, with some variations noted during the fifth hour of the experiment. Notably, films with ellagic acid dissolved in AcOH exhibited slightly higher released concentrations. Additionally, it is noteworthy that the most effective release of ellagic acid occurred in the 20HA/80EA/AcOH materials.

### 2.5. Cytocompatibility

**Figure 3:**
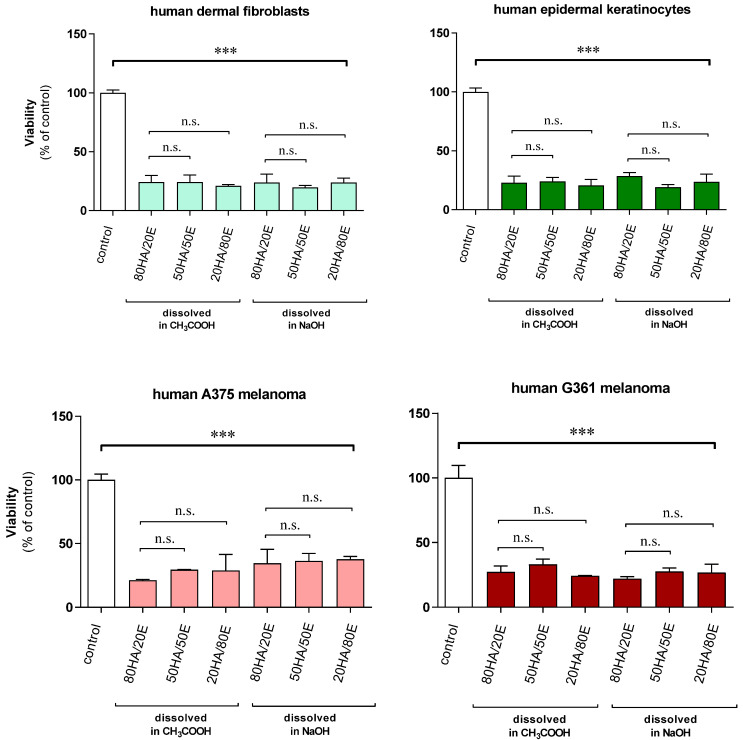
Human epidermal keratinocytes (NHEK) and dermal fibroblasts (NHDF), as well as human amelanotic melanoma cells (A375 and G-361), were seeded on respective hyaluronic acid (HA)/ellagic acid (EA)-loaded scaffolds and cultured for 96 h, and the viability was assessed using the MTT viability assay as described in Section 4. Data are presented as mean ± S.D. (*n* = 6) and expressed as a percentage of the control cells (without scaffolds). Statistically significant differences were indicated as *** *p* < 0.001; n.s.—not significant.

We assessed the impacts of solvents of ellagic acid (80HA/20EA, 50HA/50EA, and 20HA/80EA) on the viability of cutaneous cells (human dermal fibroblasts and epidermal keratinocytes) compared with human melanoma models (A375 and G361) (Figure 3). The selection of cell lines was in accordance with previous reports [25,26,27]. The obtained results demonstrated the significant decrease in the proliferation ratio versus the control cells, in both healthy and tumor cells. Specifically, the used materials caused drops in viability ranging from 72% to 80% for acetic acid and from 63% to 81% for sodium hydroxide. This decrease in cell viability indicated that the composed film had cytotoxic effects, meaning it was toxic to cells and negatively impacted their survival and proliferation. This finding suggested that a concentration of ellagic acid equal to or greater than 20% indicated cytotoxicity due to the high release concentration of ellagic acid. On the other side, there were no significant differences neither in terms of the percentage composition of the scaffolds nor in terms of the used solvent.

### 2.6. Antimicrobial Activity

**Figure 4:**
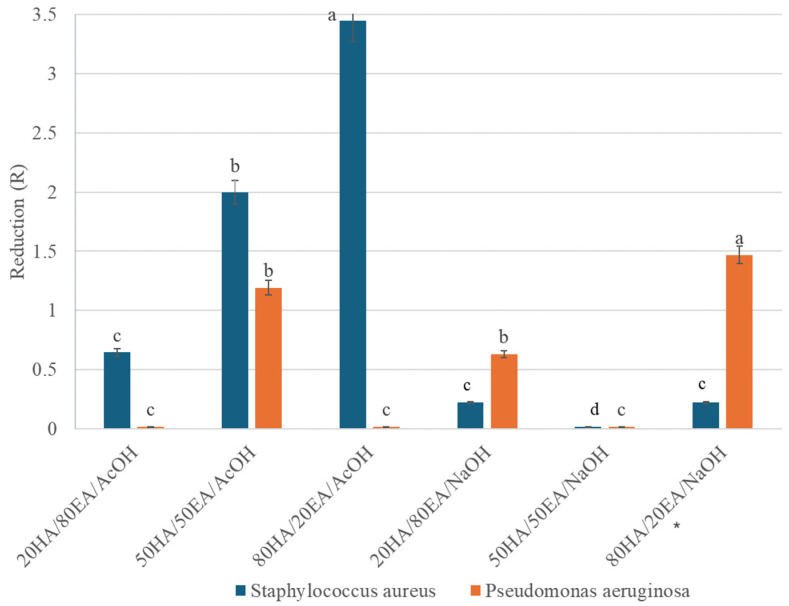
Antimicrobial activity of HA/EA. Statistical significance was tested between samples of test materials within a pathogen. Letters on the bars of the figure indicate statistically significant differences (*p* < 0.05). Values are expressed as mean ± S.D. (*n* = 3). Different letters indicate statistically significant results, and the same letters indicate no statistical significance. Statistical analysis was conducted using Past v. 3.08. The Kruskal–Wallis test and the Mann–Whitney paired test were used.

The antimicrobial properties of HA/EA (Figure 4) revealed strong biocidal properties against *Staphylococcus aureus* demonstrated by the 80HA/20EA/AcOH film where there was a 3.5-fold reduction in viable cells (R), and the biocidal activity against this strain was also demonstrated by the 50HA/50/EA/AcOH film. According to the ISO 22196:2011(E) [28], a reduction of two orders of magnitude in the number of cells capable of growing (R ≥ 2) is interpreted as a biocidal effect; however, the films tested did not have a biocidal effect against *Pseudomonas aeruginosa*. The biocidal properties were also affected by the solvent; biocidal activity was observed on films where the solvent was AcOH.

## 3. Discussion

The importance of surface roughness in thin film materials for medical applications cannot be overstated. It influences cellular morphology, proliferation, and phenotype expression. Surface roughness is classified into macroroughness (100 μm to 1 mm), microroughness (100 nm to 100 μm), and nanoroughness (<100 nm) [29,30]. Our AFM results (Table 1) indicated that the surface roughness of hyaluronic acid/ellagic acid films, regardless of the ellagic acid solvent used, fell within the microroughness range. This differed from hyaluronic acid/tannic acid films studied previously [29], which exhibited lower roughness parameters (Ra and Rq below 5 nm). The very strong hydrophilicity of the hyaluronic acid/ellagic acid films was evidenced by the immediate melting of glycerin or iodomethane drops upon application to the film surface. This hydrophilicity is consistent with the values reported for hyaluronic acid films [31] and offers advantages for potential cell interactions and biocompatibility [29,32]. Additionally, each of the materials based on HA/EA is hydrophilic since all the values of the contact angles are less than 90°. The enhanced hydrophilicity of the materials can provide additional advantages for potential cell interactions with the surface and the materials’ biocompatibility [29,32]. HA/EA films dissolved in NaOH in the same weight ratio (50HA/50EA/NaOH) were characterized by the highest hydrophilicity. Thus, it is well reported that for the combination of HA with another phenolic acid, which is tannic acid, the surface free energy decreased with the increasing amount of phenolic acid in the film’s composition [29]. Comparatively, our study showed the opposite trend, where IFT was lower for 20HA/80EA/NaOH (IFT = 50.69 ± 0.84 mJ/m^2^) than for 80HA/20EA/NaOH (IFT = 56.53 ± 0.60 mJ/m^2^) and 50HA/50EA/NaOH (IFT = 59.30 ± 0.41 mJ/m^2^) films. For instance, Kim et al. [19] evaluated the contact angle of films based on chitosan with the addition of ellagic acid, where an additional decrease in the contact angle with the increasing concentration of EA was documented. The addition of EA decreased the contact angle of the films, which is in agreement with our results for the 50HA/50EA/NaOH and 20HA/80HA/NaOH films for glycerin and the 80HA/20EA/NaOH and 20HA/80H/NaOH films for diiodomethane.

Hyaluronic acid, known as a macromolecular compound with a high ability to bind water (up to 1000 times its volume) [33], additionally exerts hydrophilic properties and the ability to swell [29]. The addition of ellagic acid to hyaluronic acid altered the moisture content of the films. As the amount of ellagic acid increased, the moisture content decreased, possibly due to residual solvent or absorption from the environment. This change is consistent with hyaluronic acid’s hygroscopic nature.

Ellagic acid, known for its potent antioxidant properties, stands in contrast to hyaluronic acid [15]. Consequently, materials exhibiting antioxidant activity hold significant medical relevance [34]. The addition of EA to HA provides antioxidant activity to the resultant films. Indeed, it was confirmed by DPPH testing of the obtained films and the exhibition of antioxidant activity by each tested material. No significant differences in antioxidant activity were observed in terms of the sample composition and the ellagic acid solvent, which may indicate only the need to introduce EA to HA in order to enhance the materials’ antioxidant characteristics.

Because hyaluronic acid/ellagic acid films exhibited antioxidant activity irrespective of the solvent used, ellagic acid release was examined for all films. The release profiles in both solvents demonstrated sustained slow release, resembling the pattern observed in chitosan/β-glycerophosphate/ellagic acid gels [35]. This sustained release is typically attributed to the gradual release of the incorporated phenolic acids from the polymer matrix, occurring during the second stage in the controlled release of EA from chitosan nanoparticles [36].

It is widely acknowledged that hyaluronic acid is non-toxic and biocompatible, with the capacity to both directly and indirectly impact the process of wound healing [37]. However, phenolic acids may show the toxicity effect with a dependence on their concentration [38]. Thereby, it is important for the cytotoxicity each material to be studied. Mohammadinejad et al. confirmed the anticancer activity of ellagic acid [39], while Kholghi et al. [40] studied the cytotoxicity of PVA-sodium alginate hydrogels containing different concentrations of EA. Their results indicated that EA nanosphere-containing hydrogels did not have any cytotoxic effect, suggesting that toxicity depended on the concentration of ellagic acid released from the material. Our studies demonstrated that the proposed materials provided a high concentration of ellagic acid, suggesting the potential benefit of reducing its content to lower amounts.

Developing natural materials with biocidal properties is crucial for medical applications. Hyaluronic acid supplemented with natural biocidal additives represents an innovative approach. Among these additives, ellagic acid holds significant promise for various biological applications. Our studies showed that the materials 50HA/50EA/AcOH and 20HA/80EA/AcOH exerted antimicrobial properties against *Staphylococcus aureus*, and this was in accordance to earlier studies where EA was reported for its antimicrobial, antioxidant properties [41], but it also inhibited the growth of methicillin-resistant *Staphylococcus aureus* and *Salmonella* [42,43]. Furthermore, Tavares et al. [20] proved the biocidal properties of EA and EA acid loaded-zein nanoparticles against *Pseudomonas aeruginosa*. Hyaluronic acid is also considered to have antibacterial, antiadhesive, bioresorbent, biodegradable, biocompatible, and immunostimulating properties [44,45,46]. An additional feature of EA is the inhibition of hyaluronidase activity, thanks to which hyaluronic acid retains its properties [47]. The hyaluronidase inhibitory activity of EA confirmed the anti-inflammatory activity and the protective effect of the extracellular matrix through the inhibition of hyaluronic acid degradation of these substances [48], while *Pseudomonas aeruginosa* and *Staphylococcus aureus* are opportunistic pathogens that are most commonly co-isolated from chronic wounds [49]. Altogether, HA/EA with its biocidal properties against these bacteria offers numerous chances for dermatological applications.

## 4. Materials and Methods

### 4.1. Materials

Hyaluronic acid sodium salt (HA, M = 1.8 × 10^6^ g/mol) and ellagic acid (EA, M = 302.20 g/mol, anhydrous) were purchased from Pol-Aura company (Poznań, Poland). Diiodomethane (99%), Minimum Essential Medium Eagle (MEM) (1000 mg/L), 1% penicillin-streptomycin solution, 0.05% trypsin/0.53 mM EDTA solution, 3-(4,5-dimethylthiazol-2-yl)-2,5-diphenyltetrazolium bromide (MTT), ethanol (EtOH), HCl, *L*-glutamine (200 mM), and isopropanol were purchased from Sigma (St. Louis, MO, USA), while glycerin was purchased from Avantor Performance Materials Poland S.A. (Gliwice, Poland). Fetal bovine serum was supplied by Thermo Fisher Scientific (Waltham, MA, USA). Plate count agar (PCA) for bacterial culture was from Biocorp (Warsaw, Poland). Titration plates for the determination of antimicrobial activity and sterile plates for bacterial culture were from CytoGen (Zgierz, Poland).

### 4.2. Film Preparation

HA was dissolved in deionized water at a 1% (*w*/*v*) concentration, while EA was dissolved at a 4% (*w*/*v*) concentration in 0.0015 M NaOH or in 0.1 M AcOH, accordingly. AcOH and NaOH were selected as solvents for ellagic acid because they were also miscible with the chitosan solution. HA and EA solutions were mixed in three different weight ratios as follows: 80HA/20EA, 50HA/50EA, and 20HA/80EA. The solutions (40 mL) were mixed by the magnetic stirrer for 1 h in room conditions (temperature, humidity, 400 r.p.m.) and placed on the plastic holder (10 × 10 cm). The designations and compositions of the obtained materials are presented accordingly (Table 4).

### 4.3. Atomic Force Microscopy

The images of the films’ surfaces were obtained with the microscope equipped with a scanning SPM probe of the NanoScope MultiMode type (Veeco Metrology, Inc., Santa Barbara, CA, USA) operated in a tapping mode (room conditions; a scan rate of 1.97 Hz; a spring constant 2–10 N/m of silicon tips). Then, within the Nanoscope v6.11 software (Bruker Optoc GmbH, Ettlingen, Germany), two roughness parameters were calculated: the root-mean-square (Rq) and roughness and the arithmetic mean (Ra).

### 4.4. Surface Free Energy

The surface free energy (IFT (s)) and its polar (IFT (s,P)) and dispersive (IFT (s,D)) components were calculated using the Owens–Wendt method. For that purpose, the contact angles were measured using two liquids (glycerin and diiodomethane) in a constant temperature value, using a goniometer equipped with a drop shape analysis system (DSA 10 Control Unit, Krüss, Hamburg, Germany).

### 4.5. Moisture Content

Moisture content analysis was performed using a Radwag MA 50.X2.IC.A moisture analyzer. Briefly, the sample (4 × 4 cm) was placed on a pan, weighed, and dried at 105 °C to a constant mass, and the moisture content (MC) was calculated.

### 4.6. Antioxidant Activity

The antioxidant activity of the films was determined using the DPPH reagent (2,2-diphenyl-1-picrylhydrazyl, free radical, 95%; Alfa Aesar, Karlsruhe, Germany). Samples (1 × 1 cm) of each film were placed in a 24-well plate, filled with DPPH solution (2 mL, 250 µM solution in methyl alcohol), and kept for 1 h in darkness. A spectrophotometric assessment was performed using the UV-1800 (Shimadzu, Muttenz, Switzerland) at 527 nm. The antioxidant activity was calculated as follows:

(1) $$RSA\%=\frac{{Abs}_{DPPH}-{Abs}_{PB}}{{Abs}_{DPPH}}\times 100\%$$

where *Abs_DPPH_* is the absorbance of the DPPH solution without contact with the material being tested, and *Abs_PB_* is the absorbance of the DPPH solution after contact with the material being tested.

### 4.7. Phenolic Acid Release

The EA release study was carried out in PBS (pH = 7.4) using the Folin–Ciocalteu method [50] where tested samples were downloaded every 1, 2, 3, 4, and 5 h. To perform this analysis, 0.5 mL of sample was mixed with 1 mL of Na_2_CO_3_ and 0.5 mL of Folin–Ciocalteu reagent. Then, the mixture was filled by distilled water up to 10 mL and stored in 40 °C for 30 min. Afterward, the absorbance was measured at 725 nm using the UV–Vis spectrophotometer (UV-1800, Shimadzu, Switzerland).

### 4.8. Cell Culture

Normal human epidermal keratinocytes (NHEKs) and normal human dermal fibroblasts (NHDFs) were supplied by PromoCell (Heidelberg, Germany) and American Type Culture Collection (ATCC) (Manassas, VA, USA), respectively. NHEKs were grown in Keratinocyte Growth Medium 2 supplemented with 1% penicillin–streptomycin solution, while NHDFs were maintained in MEM medium supplemented with 10% (*v*/*v*) heat-inactivated fetal bovine serum, 2 mM *L*-glutamine, and 1% (*v*/*v*) streptomycin–penicillin solution. Comparatively, human melanoma cell lines, i.e., amelanotic A375 (CRL-1619) and G-361 (CRL-1424), were supplied by ATCC (Manassas, VA, USA). Cells were maintained in MEM medium supplemented with 10% (*v*/*v*) heat-inactivated fetal bovine serum, 2 mM *L*-glutamine, and 1% (*v*/*v*) streptomycin–penicillin solution. Cells were seeded on 24-well plates at the density of 0.5 × 10^5^ cells/well and were allowed to attach to the surface of the subjected scaffolds for 24 h. After that, the cells were cultured in supplemented culture medium in a humidified atmosphere of 5% CO_2_ at 37 °C for 96 h while the culture medium was exchanged every 48 h. Differences in cell viability were assessed using the MTT assay.

### 4.9. Cell Viability Assay

MTT (5 mg/mL in 1 × PBS) was prepared in its respective culture medium (the final dilution, 1:10), 100 μL of assay reagent was added to each well, and cells were subsequently incubated for 3 h in a humidified atmosphere of 5% CO_2_ at 37 °C. The resultant formazan crystals were dissolved using 100 μL isopropanol/0.04 N HCl, the absorbance was measured at *λ* = 595 nm using the BioTek ELx808™ microplate reader (BioTek Instruments, Inc., Winooski, VT, USA), and the results were normalized to the control cells.

### 4.10. Antimicrobial Activity

The antibacterial activity of 100HA, 80HA/20EA/AcOH, 50HA/50EA/AcOH, 20HA/80EA/AcOH, 80HA/20EA/NaOH, 50HA/50EA/NaOH, and 20HA/80EA/NaOH was evaluated against *Staphylococcus aureus* ATCC 6538 and *Pseudomonas aeruginosa* ATCC15442. The bacterial inoculum was prepared by suspending the bacterial colony from an overnight culture on plate count agar (PCA) medium in 10 mL nutrient broth. The suspension was diluted to the final cell concentration of 10^8^ CFU/mL, confirmed additionally by plating on PCA. The materials (2 × 2 cm) were placed in a tetrahedral titration plate, and 0.2 mL of bacterial inoculum was added. Finally, the resultant films were covered with sterile parafilm. The tetrahedral titration plates were incubated at 37 °C for 24 h, and after incubation, the numbers of bacteria were assessed by plating cell suspensions in PCA. Next, the plates were incubated at 37 °C for 24 h, and the resulting colonies were counted accordingly. The reduction in microbial counts (R) was determined according to ISO 22196:211 (E) [28] using the formula

(2) $$R=\left(U_{t}-U_{0}\right)-\left(A_{t}-U_{0}\right)=U_{t}-A_{t}$$

where

*R*—antimicrobial activity;
*U_0_*—average of logarithm numbers of viable bacteria from the control sample at time = 0 h;
*U_t_*—average of logarithm numbers of viable bacteria from the control sample at time = 24 h;
*A_t_*—average of logarithm numbers of viable bacteria from the test sample at time = 24 h.

### 4.11. Statistical Analysis

Statistically significant differences between the results were determined by the univariate analysis of variance (ANOVA) or the Student’s *t*-test and appropriate post hoc analysis. Multiple comparisons between the means were performed with the statistical significance set at *p* ≤ 0.05. Experiments were carried out at least in triplicates (*n* ≤ 3). Tukey’s pairwise test was applied for post hoc comparisons. Statistical assessments were carried out using GraphPad Prism 8.0.1.244 software (GraphPad Software, San Diego, CA, USA).

## 5. Conclusions

The study provides insights into films made of hyaluronic acid (HA) and ellagic acid (EA) for biomedical applications, particularly wound dressing. Variations in surface roughness were observed based on the composition and the solvent used for EA dissolution. Changes in wettability were noted, with higher EA content leading to lower hydrophilicity. The films with HA/EA/NaOH had lower moisture content compared with the HA/EA films. Positive radical scavenging activity was observed in all HA/EA films. Ellagic acid release profiles remained consistent across different solvents. While the films showed cytotoxic effects on cell proliferation, no significant differences were noted based on composition or solvent. It may be assumed that the proposed materials could be utilized for anticancer therapy as functional platforms that create an inhospitable environment for the growth of cancer cells. However, to deploy them in medicine, the ellagic acid content should be reduced.

## Figures and Tables

**Table 1:** Roughness parameters of HA/EA films. Ra—the arithmetic mean roughness; Rq—the root-mean-square roughness; * significant difference versus 80HA/20EA/AcOH; ** significant difference versus 20HA/80EA/NaOH; n.s.—not significant. Roughness analyses were calculated from 100 μm^2^ surface area.

Specimen	Ra [nm]	Rq [nm]
80HA/20EA/AcOH	369 ± 27	297 ± 23
50HA/50EA/AcOH	273 ± 35	217 ± 27
20HA/80EA/AcOH	320 ± 45	260 ± 36
80HA/20EA/NaOH	260 ± 32 *	204 ± 26 ^n.s.^
50HA/50EA/NaOH	297 ± 37 ^n.s.^	240 ± 28 ^n.s.^
20HA/80EA/NaOH	472 ± 21 **	385 ± 16 **

**Table 2:** The results of contact angles, surface free energy (IFT (s)), and its dispersive (IFT (s,D)) and polar (IFT (s,P)) components of the HA/EA films (*n* = 5; * significant difference versus control sample (100HA) (*p* < 0.05); n.s.—not significant; G—glycerine; D—diiodomethane).

Specimen	Contact Angle [°]		IFT (s) [mJ/m^2^]	IFT (s,D) [mJ/m^2^]	IFT (s,P) [mJ/m^2^]
	G	D			
100HA	35.28 ± 2.29	58.16 ± 1.46	51.71 ± 0.74	17.44 ± 0.26	34.28 ± 0.48
80HA/20EA/AcOH	Measurement impossible				
50HA/50EA/AcOH					
20HA/80EA/AcOH					
80HA/20EA/NaOH	37.70 ± 1.67 ^n.s.^	51.33 ± 2.07 *	50.69 ± 0.84	21.48 ± 0.41	29.21 ± 0.43
50HA/50EA/NaOH	23.83 ± 1.10 *	68.95 ± 0.83 *	59.30 ± 0.41	10.93 ± 0.12	48.37 ± 0.29
20HA/80EA/NaOH	26.24 ± 1.16 *	57.90 ± 1.43 ^n.s.^	56.53 ± 0.60	16.66 ± 0.25	39.86 ± 0.35

**Table 3:** The results of moisture content (MC) and radical scavenging activity (RSA) relevant to antioxidant activity of the HA/EA films (*n* = 3; * significant difference versus control sample (100HA) (*p* < 0.05)).

Specimen	MC [%]	RSA [%]
100HA	15.26 ± 0.99	−21.50 ± 0.02
80HA/20EA/AcOH	8.94 ± 0.61 *	85.40 ± 0.01 *
50HA/50EA/AcOH	9.65 ± 1.16 *	84.80 ± 0.01 *
20HA/80EA/AcOH	6.40 ± 0.38 *	85.11 ± 0.01 *
80HA/20EA/NaOH	12.50 ± 0.93 *	86.23 ± 0.06 *
50HA/50EA/NaOH	4.60 ± 0.52 *	83.21 ± 0.07 *
20HA/80EA/NaOH	3.27 ± 0.35 *	85.15 ± 0.04 *

**Table 4:** Compositions of the obtained materials: hyaluronic acid (HA), ellagic acid (EA), acetic acid (AcOH), and sodium hydroxide (NaOH).

Sample	HA Content [wt.%]	EA Content [wt.%]	EA Solvent
100HA	100	-	-
80HA/20EA/AcOH	80	20	0.1 M AcOH
50HA/50EA/AcOH	50	50	
20HA/80EA/AcOH	20	80	
80HA/20EA/NaOH	80	20	0.0015 M NaOH
50HA/50EA/NaOH	50	50	
20HA/80EA/NaOH	20	80	

## Data Availability

Data is contained within the article.
